# Validity of a Self-administered Food Frequency Questionnaire Used in the 5-year Follow-up Survey of the JPHC Study Cohort I to Assess Carotenoids and Vitamin C Intake: Comparison with Dietary Records and Blood Level

**DOI:** 10.2188/jea.13.1sup_82

**Published:** 2007-11-30

**Authors:** Minatsu Kobayashi, Satoshi Sasaki, Shoichiro Tsugane

**Affiliations:** 1 Epidemiology and Biostatistics Division, National Cancer Center Research Institute East.

**Keywords:** carotenoid, vitamin C, validity, food frequency questionnaire, dietary record, blood

## Abstract

We compared carotene and vitamin C intake assessed with our 138-item food frequency questionnaire (FFQ) against 28-day weighed dietary records among a subgroup of JPHC Study Cohort I (102 men and 113 women), and the corresponding serum carotenoid levels or plasma vitamin C levels (86 men and 100 women). Correlation coefficients between carotenoids or vitamin C intake estimated from FFQ and intakes estimated from DR were as follows in men and women, respectively: alpha-carotene, r=0.47 and r=0.46; beta-carotene, r=0.40 and r=0.30; lycopene, r=0.18 and r=0.22; vitamin C, r=0.44 and r=0.31. Correlation coefficients between carotenoids or vitamin C intake estimated from FFQ and the corresponding serum carotenoids levels or plasma vitamin C levels were as follows: alpha-carotene, r=0.38 and r=0.30; beta-carotene, r=0.28 and r=0.11; lycopene, r=0.30 and r=0.19; vitamin C, r=0.07 and r=0.06; in men and women, respectively. These data indicated carotenoid and vitamin C intakes estimated from FFQ were associated with intake from DR, although the association was weak for lycopene. Carotenoid intake estimated from FFQ were associated with corresponding serum carotenoid levels in men, but the correlation was weak in women except for alpha-carotene. Both in men and women, no association was observed for plasma vitamin C levels.

Several epidemiological studies have provided the evidence for an inverse association of vegetable and fruit intake with certain cancers.^1^^-^^3^ The beneficial effect of vegetable and fruit could be partly due to antioxidant nutrients such as carotenoids or vitamin C, which might protect biological structures from oxidative damage and reduce lipid peroxidation.^4^ In Japan, until recently no comprehensive food composition database for individual carotenoids has been available, making it difficult to assess the possible relationship between specific carotenoid intake and the risk of disease.

In the present study, we estimated the alpha-carotene, beta-carotene and lycopene intake from both our 138-item food frequency questionnaire (FFQ) and 28-day weighed dietary records (DR) among a subgroup of JPHC Study Cohort I using our newly-developed food composition database for individual carotenoids.^5^ We compared the carotenoid intake and vitamin C intake estimated from both the FFQ and DR with the corresponding serum carotenoid level or plasma vitamin C level.

## MATERIALS AND METHODS

The study design and subject characteristics have been reported elsewhere in this Supplement.^6^ The methods for surveying dietary records and for computing nutrient intakes from FFQ have also been described therein.^7^^,^^8^

### Carotenoid and Vitamin C Intake Calculations

The daily intakes of total carotenoids and vitamin C were calculated using the Standard Tables of Food Composition in Japan.^9^ The daily intakes of specific carotenoids, such as alpha-carotene, beta-carotene and lycopene, were calculated using our new carotene database.^5^

### Laboratory Measurements

The details of blood collection have been presented elsewhere in this report.^6^ Briefly, the blood was collected just before winter or just after summer 7-day DR. The plasma and serum samples were stored in separate tubes and stored at -80°C until analysis. Plasma collected in February. was stabilized by metaphosphoric acid for ascorbic acid measurement.

Serum carotenoids were quantified by high-performance liquid chromatography (HPLC) using a previously-described modified method.^10^ Briefly, the components were extracted by shaking and mixing for 30 min after addition of each of the following: 250 *µ*L of water, 500 *µ*L of ethyl alcohol, 4 mL of *n*-hexane, and 400 *µ*L of serum. After centrifugation, the *n*-hexane layer was dried with N_2_ gas. To determine carotenoids (alpha-carotene, beta-carotene, lycopene), the residue was dissolved in 30 *µ*L of dichloromethane and 200 *µ*L of the mixed solvent. Then, 200 *µ*L of the solution was applied to a HPLC (Waters, Milford, Mass) with the following conditions: C18 reverse phase column, Wakopak (Wako, Osaka, Japan); mobile phase, methanol: acetonitrile: water=60:40:1; UV wavelength, 450; flow rate, 1.0 ml/min.

Plasma ascorbic acid was measured by spectrophotometer using a modified method described previously.^11^ Briefly, 450 *µ*L of the samples was sequentially mixed with 0.15 mL of 0.15% dithiothreitol, 0.15 mL of 0.5% N-ethylmaleimide, and 0.75 mL of trichloroacetic acid. After centrifugation, the supernatant was mixed with 0.75 mL of a chromogen (phosphate: water: 1.8%FeCl_3_: 4%dipyridyl=1:1:1:2), incubated at 37C° for 30 min, and then the optical density at 525 nm was measured with a UV/Vis spectrophotometer (V-550, Nihon Bunko, Tokyo, Japan).

### Statistical Analysis

The subjects of this study were 215 persons (102 men and 113 women). Both their FFQ for the validation study and the complete DR (14-day records in Ishikawa PHC area and 28-day records on other 3 areas) were included in this analysis. As for the analysis using the serum carotenoid level, 186 subjects (86 men and 100 women) were measured twice (Feb. and Aug.). For their plasma ascorbic acid use, 185 subjects (86 men and 99 women) were measured (Feb.).

The mean, SD and median intakes for carotenoids (alpha-carotene, beta-carotene, lycopene) and vitamin C from the FFQ and DRs were calculated by sex and area. The Spearman rank correlation was used to assess the association between carotenoid and vitamin C intake from FFQ and from DR. Serum carotenoids and plasma vitamin C were presented as mean, SD and median by sex and area. The Spearman correlation was again used, this time to examine the association between intakes (carotenoids and vitamin C assessed by FFQ or DR) and blood levels (serum carotenoids and plasma ascorbic acid). The mean intake of carotenoids and vitamin C assessed by DR and the mean biochemical indicator concentrations were calculated according to quintile of intake assessed with FFQ. All statistical analyses were performed using the SAS statistical software package.^12^

## RESULTS

Table 1 shows carotenoid and vitamin C intakes by sex and area estimated from the DR. In men, alpha-carotene and beta-carotene intakes were highest in the Ishikawa PHC area. Lycopene intakes were highest in the Ninohe PHC area. In women, no apparent area-difference was observed for carotenoid intake except that alpha-carotene intake was the highest in the Ishikawa PHC area. No apparent area-difference was observed for vitamin C intakes both in men and women.

**Table 1.** Carotenoid intakes (*µ*g/day) and vitamin C intakes (mg/day) assessed with DR^1^ for 28 or 14 days by area

	Ninohe PHC				Yokote PHC				Saku PHC				Ishikawa PHC				ANOVA p-value
				
	Mean	±	SD	Median	Mean	±	SD	Median	Mean	±	SD	Median	Mean	±	SD	Median	
Men (n=102)	n=24				n=28				n=23				n=27				
Carotene	3564	±	1330	3304	2503	±	798	2441	3174	±	874	2972	3902	±	1616	3919	0.0003
Alpha-carotene	507	±	251	446	286	±	115	271	352	±	152	308	669	±	333	652	0.0001
Beta-carotene	2726	±	1055	2476	2021	±	697	1919	2578	±	724	2516	3111	±	1310	3007	0.0011
Lycopene	4336	±	4230	3042	2515	±	1551	1966	2456	±	1342	2292	2622	±	2531	1844	0.0414

Vitamin C	122	±	38	115	122	±	34	123	139	±	40	134	134	±	45	130	0.3083

Women (n=113)	n=27				n=30				n=28				n=28				
Carotene	3314	±	1471	3126	2773	±	941	2658	3109	±	972	2837	3573	±	1505	3746	0.1000
Alpha-carotene	446	±	236	359	337	±	169	288	358	±	142	319	581	±	260	571	0.0001
Beta-carotene	2597	±	1218	2386	2241	±	766	2038	2514	±	807	2332	2860	±	1241	2888	0.1537
Lycopene	395	±	3004	3241	2832	±	1894	2125	3243	±	2262	2628	3261	±	3412	2085	0.4721
	
Vitamin C	128	±	44	114	133	±	36	127	146	±	46	138	139	±	71	117	0.5870

^1^DR, dietary records.

Table 2 shows carotenoid and vitamin C intakes by sex and area estimated from FFQ. No apparent area-difference was observed for any carotenoid and vitamin C intakes in either men or women.

**Table 2.** Carotenoid intakes (*µ*g/day) and vitamin C intakes (mg/day) assessed with FFQ^1^ by area

	Ninohe PHC				Yokote PHC				Saku PHC				Ishikawa PHC				ANOVA p-value
				
	Mean	±	SD	Median	Mean	±	SD	Median	Mean	±	SD	Median	Mean	±	SD	Median	
Men (n=102)	n=24				n=28				n=23				n=27				
Carotene	3722	±	2816	3588	3719	±	4606	2346	4120	±	1818	3551	3736	±	2450	3308	0.9643
Alpha-carotene	607	±	661	491	449	±	636	228	523	±	382	403	669	±	441	618	0.4754
Beta-carotene	2891	±	2168	2762	3015	±	3923	2022	3365	±	1444	3126	2935	±	1965	2377	0.9230
Lycopene	3288	±	5203	816	4543	±	5368	2761	3407	±	3478	2345	2254	±	4075	437	0.3424

Vitamin C	146	±	95	115	198	±	182	170	192	±	65	196	130	±	68	111	0.0910

Women (n=113)	n=27				n=30				n=28				n=28				
Carotene	3670	±	2787	2669	3867	±	4194	2549	4638	±	2664	4268	4247	±	2007	3928	0.6496
Alpha-carotene	513	±	485	350	486	±	627	241	563	±	421	471	759	±	377	635	0.1515
Beta-carotene	2906	±	2261	2187	3140	±	3524	2176	3769	±	2129	3515	3342	±	1605	2986	0.6215
Lycopene	2762	±	2566	1439	4262	±	6796	2502	4212	±	5993	2049	1261	±	1743	535	0.0676

Vitamin C	172	±	106	143	208	±	229	141	244	±	164	203	142	±	71	115	0.0915

^1^FFQ, food frequency questionnaire.

Table 3 shows mean and median serum carotenoid and plasma vitamin C levels by sex and area. In men, the serum beta-carotene level was highest in the Saku PHC area. In women, the serum alpha-carotene level was highest in the Ishikawa PHC area and the serum beta-carotene level was highest in the Saku PHC area. Both in men and women, the plasma vitamin C level was highest in the Ninohe PHC area.

**Table 3.** Serum carotenoid level^1^ (mg/ml) and plasma vitamin C level^1^ (mg/dl) by area

	Ninohe PHC				Yokote PHC				Saku PHC				Ishikawa PHC				ANOVA p-value
				
	Mean	±	SD	Median	Mean	±	SD	Median	Mean	±	SD	Median	Mean	±	SD	Median	
Men (n=86)	n=22				n=25				n=18				n=21				
Alpha-carotene	0.06	±	0.04	0.05	0.05	±	0.02	0.05	0.05	±	0.02	0.05	0.07	±	0.03	0.06	0.2226
Beta-carotene	0.30	±	0.2	0.21	0.26	±	0.1	0.24	0.37	±	0.23	0.32	0.21	±	0.07	0.20	0.0230
Lycopene	0.08	±	0.03	0.08	0.10	±	0.03	0.09	0.10	±	0.04	0.09	0.09	±	0.02	0.08	0.2307

Vitamin C	1.36	±	0.27	1.38	1.07	±	0.24	1.01	1.24	±	0.22	1.24	1.08	±	0.22	1.08	0.0002

Women (n=100)	n=26				n=26				n=24				n=24				
Alpha-carotene	0.06	±	0.02	0.06	0.07	±	0.03	0.07	0.08	±	0.04	0.08	0.11	±	0.05	0.11	0.0001
Beta-carotene	0.40	±	0.16	0.38	0.46	±	0.27	0.36	0.68	±	0.45	0.55	0.42	±	0.19	0.35	0.0037
Lycopene	0.10	±	0.03	0.09	0.10	±	0.04	0.08	0.11	±	0.04	0.10	0.10	±	0.02	0.10	0.3502

Vitamin C^2^	1.53	±	0.33	1.54	1.16	±	0.29	1.22	1.40	±	0.22	1.36	1.17	±	0.21	1.13	0.0001

^1^Serum carotenoid level, mean measurments of Feb. and Aug.; Plasma vitaminC level, measured in Feb.

^2^for Ishikawa PHC, n=23

Table 4 shows intake levels of carotenoids and vitamin C estimated from DR and FFQ in 4 areas. Both in men and women, total carotene, beta-carotene, lycopene and vitamin C intakes estimated from FFQ were higher than intake from DR except for lycopene in women. Table 4 also presents the relation between carotenoid and vitamin C intake estimated from FFQ, and carotenoids and vitamin C intake estimated from DR. Spearman rank correlation coefficients between carotenoid or vitamin C intake estimated from FFQ and intakes estimated from DR were as follows: alpha-carotene, r=0.47 and r=0.46; beta-carotene, r=0.40 and r=0.30; lycopene, r=0.18 and r=0.22; vitamin C, r=0.44 and r=0.31; in men and women, respectively. No improvement in the correlation coefficient was observed when intakes were expressed as energy-adjusted value.

**Table 4.** Carotenoid intakes (*µ*g/day) and vitamin C intakes (mg/day) assessed with DR^1^ for 28 or 14 days and FFQ^2^ in 4 areas and correlations

Sex	DR				FFQ				% difference	Spearman correlation	
		
	Mean	±	SD	Median	Mean	±	SD	Median		Crude	Energy-adjusted^3^
Men (n=102)											
Carotene	3274	±	1305	2885	3814	±	3126	3320	16	0.38	0.36
Alpha-carotene	454	±	273	357	561	±	545	398	23	0.47	0.47
Beta-carotene	2601	±	1052	2314	3044	±	2582	2556	17	0.40	0.41
Lycopene	2965	±	2715	2121	3386	±	4636	1296	14	0.18	0.19

Vitamin C	129	±	39	127	166	±	118	157	29	0.44	0.42

Women (n=113)											
Carotene	3184	±	1262	2870	4105	±	3029	3358	29	0.31	0.33
Alpha-carotene	429	±	226	348	579	±	495	433	35	0.46	0.50
Beta-carotene	2547	±	1037	2344	3290	±	2493	2789	29	0.30	0.32
Lycopene	3309	±	2689	2450	3148	±	4942	1438.86	-5	0.22	0.11

Vitamin C	137	±	50	127	192	±	159	156	41	0.31	0.22

^1^DR, dietary records.

^2^FFQ, food frequency questionnaire.

^3^Energy was adjusted by residual model for intake.

For n=102, r>0.20 = p<0.05, r>0.26 = p<0.01, r>0.33 = p<0.001.

For n=113, r>0.19 = p<0.05, r>0.25 = p<0.01, r>0.31 = p<0.001.

Table 5 shows the mean carotenoid and vitamin C intake estimated from DR within quintiles of corresponding carotenoid and vitamin C intake estimated from FFQ. In men, the mean intake in the highest quintile was 1.5 times higher or more than in the lowest quintile for any carotenoid and vitamin C. In women, only alpha-carotene was more than 1.5 times higher. A steady increase in mean intake from the lowest to the highest quintile was observed for alpha-carotene in men and beta-carotene in women. The result of cross-classification of the subjects by quintiles from carotenoids and vitamin C intake from DR and carotenoids and vitamin C intake from FFQ are shown in Table 6. Both in men and women, more than 60% of them were classified in the adjacent quintiles and less than 6% of them were classified in the extreme quintiles.

**Table 5.** Mean intake of carotenoids and vitamin C from DR^1^ within quintile of intake determined by FFQ^2^

	Quintile of carotenoids and vitamin C intake according to FFQ																		

Sex Carotenoids	Lowest			Second				Third				Fourth				Highest			
				
	Mean	±	SD	Mean	±	SD	ratio^3^	Mean	±	SD	ratio^3^	Mean	±	SD	ratio^3^	Mean	±	SD	ratio^3^
Men	n=20			n=21				n=20				n=21				n=20			
Carotene	2541	±	950	3067	±	1185	1.21	3528	±	1591	1.39	3365	±	1115	1.32	3877	±	1329	1.53
Alpha-carotene	320	±	197	331	±	147	1.04	477	±	266	1.49	538	±	349	1.68	608	±	260	1.90
Beta-carotene	2073	±	692	2329	±	954	1.12	2796	±	1292	1.35	2602	±	791	1.26	3219	±	1140	1.55
Lycopene	2423	±	1812	2801	±	2895	1.16	2626	±	1761	1.08	2823	±	2080	1.16	4115	±	4140	1.70

Vitamin C	103	±	30	130	±	42	1.26	120	±	27	1.17	134	±	36	1.30	158	±	41	1.54

Women	n=22			n=23				n=23				n=23				n=22			
Carotene	2604	±	1012	3021	±	1092	1.16	3142	±	1169	1.21	3581	±	1624	1.37	3561	±	1151	1.37
Alpha-carotene	319	±	197	356	±	169	1.12	456	±	227	1.43	450	±	220	1.41	565	±	243	1.77
Beta-carotene	2092	±	822	2489	±	841	1.19	2556	±	1200	1.22	2732	±	1189	1.31	2860	±	983	1.37
Lycopene	3329	±	3641	2726	±	2303	0.82	3617	±	3065	1.09	3068	±	2109	0.92	3831	±	2102	1.15

Vitamin C	122	±	44	127	±	65	1.04	138	±	43	1.13	145	±	44	1.20	151	±	52	1.24

^1^DR, dietary records.

^2^FFQ, food frequency questionnaire.

^3^Ratio compared to lowest quintile.

**Table 6.** Comparison of FFQ^1^ with DR^2^ for carotenoids and vitamin C based on joint classification by quintile (%)

Carotenoids	Men (n=102)			Women (n=113)		
	
	Same category	Adjacent category	Extreme category	Same category	Adjacent category	Extreme category
Carotene	30	71	4	24	63	3
Alpha-carotene	29	71	3	25	70	2
Beta-carotene	30	69	5	24	60	4
Lycopene	17	61	6	27	62	5

Vitamin C	36	73	1	26	66	4

^1^FFQ, food frequency questionnaire.

^2^DR, dietary records.

Table 7 shows the mean, SD and median serum carotenoid levels and plasma vitamin C level in 4 areas. All serum carotenoid and plasma vitamin C levels were higher in women than in men. Table 7 also presents the relation between serum carotenoid level or plasma vitamin C level and corresponding carotenoids or vitamin C intakes estimated from FFQ and DR. In men, serum carotenoid levels were moderately correlated with the respective intake estimated from both the DR and FFQ. Correlation coefficients were as follows: alpha-carotene, both r=0.38; beta-carotene, r=0.28 and r=0.21; lycopene, r=0.30 and r=0.28; from FFQ and DR, respectively. In women, a moderate correlation was observed between serum carotenoid levels and the respective intake estimated from DR: alpha-carotene, r=0.40; beta-carotene, r=0.26; lycopene, r=0.34. However, this correlation was not observed with intake estimated from FFQ except for alpha-carotene: alpha-carotene, r=0.30; beta-carotene, r=0.11; lycopene, r=0.19. As for the plasma vitamin C, no significant correlation was observed either in men (r=-0.07, r=-0.01 for FFQ and DR, respectively) or women (r=0.06, r=0.07 for FFQ and DR, respectively). No improvement in the correlation coefficient was observed when intakes were expressed as an energy-adjusted value.

**Table 7.** Serum Carotenoid (mg/ml) and plasma vitamin C (mg/dl) in 4 areas and correlations with carotenoid intakes, vitamin C intakes assessed with either DR^1^ and FFQ^2^

Sex				Spearman correlation			
	
Carotenoids				DR		FFQ	
		
	Mean	SD	Median	crude	energy - adjusted^3^	crude	energy - adjusted^3^
Men (n=86)							
Total carotene	0.35	0.25	0.27	0.25	0.25	0.30	0.21
Alpha-carotene	0.06	0.03	0.05	0.38	0.38	0.38	0.34
Beta-carotene	0.29	0.23	0.22	0.21	0.20	0.28	0.18
Lycopene	0.09	0.03	0.08	0.28	0.34	0.30	0.26

Vitamin C	1.17	0.27	1.13	-0.01	-0.13	-0.07	-0.20

Women (n=100)^4^							
Total carotene	0.57	0.33	0.50	0.25	0.26	0.12	0.08
Alpha-carotene	0.08	0.04	0.07	0.40	0.43	0.30	0.30
Beta-carotene	0.49	0.31	0.41	0.26	0.25	0.11	0.03
Lycopene	0.10	0.04	0.09	0.34	0.37	0.19	0.10

Vitamin C	1.31	0.31	1.32	0.07	-0.02	0.06	-0.14

^1^DR, dietary records.

^2^FFQ, food frequency questionnaire.

^3^Energy was adjusted by residual model for intake.

^4^For vitamin C, women (n=99).

For n=86, r>0.22 = p<0.05, r>0.28 = p<0.01, r>0.35 = p<0.001.

For n=100, r>0.20 = p<0.05, r>0.26 = p<0.01, r>0.33 = p<0.001.

Table 8 shows the mean serum carotenoid and plasma vitamin C levels within quintiles of corresponding carotenoid and vitamin C intake estimated from FFQ. The ratio of the mean intake in the highest to the lowest quintile in men was higher than in women. The result of cross-classification of the subjects by quintiles from serum carotenoid level or plasma vitamin C level and carotenoids and vitamin C intake from FFQ are shown in Table 9. Both in men and women, more than 50% of the subjects were classified in the same or adjacent quintiles, and less than 10% of the subjects were classified in the extreme quintiles.

**Table 8.** Serum carotenoid and plasma vitamin C within quintile of carotenoid and vitamin C intake from FFQ^1^

	Quintile of serum carotenoid and plasma vitamin C level																	

Carotenoid	Lowest			Second				Third				Fourth				Highest			
				
	Mean	±	SD	Mean	±	SD	ratio^2^	Mean	±	SD	ratio^2^	Mean	±	SD	ratio^2^	Mean	±	SD	ratio^2^
Men (n=86)																		
Total carotene	2677	±	1598	4023	±	2277	1.50	4718	±	6072	1.76	3655	±	1804	1.37	6847	±	4212	2.56
Alpha-carotene	348	±	243	432	±	337	1.24	793	±	811	2.28	543	±	296	1.56	1221	±	1219	3.51
Beta-carotene	2283	±	1791	3060	±	1544	1.34	4172	±	5109	1.83	2450	±	994	1.07	5366	±	3007	2.35
Lycopene	1819	±	2763	2702	±	3618	1.49	2441	±	3151	1.34	4687	±	5732	2.58	3973	±	4520	2.18

Vitamin C	191	±	192	165	±	96	0.87	175	±	70	0.92	125	±	55	0.66	168	±	97	0.88

Women (n=100)^3^																		
Total carotene	3844	±	2388	3070	±	1817	0.80	4865	±	4549	1.27	3900	±	2553	1.01	4152	±	2633	1.08
Alpha-carotene	402	±	336	380	±	285	0.95	604	±	717	1.50	725	±	532	1.81	622	±	406	1.55
Beta-carotene	2783	±	2004	2978	±	2010	1.07	3819	±	3885	1.37	2984	±	1766	1.07	3377	±	2144	1.21
Lycopene	2166	±	2027	2361	±	2988	1.09	3801	±	7273	1.76	4697	±	7399	2.17	2798	±	2440	1.29

Vitamin C	156	±	61	154	±	57	0.99	185	±	84	1.18	264	±	282	1.69	184	±	107	1.18

^1^FFQ, food frequency questionnaire.

^2^Ratio compared to the lowest quintile.

^3^For vitamin C, women (n=99).

**Table 9.** Comparison of serum carotenoids and plasma ascorbic acid with FFQ^1^ for intakes of carotenoids and vitamin C based on joint classification by quintile (%)

Carotenoids	Men (n=86)			Women (n=100)^2^		
	
	Same category	Adjacent category	Extreme category	Same category	Adjacent category	Extreme category
Total carotene	24	60	1	23	52	6
Alpha-carotene	23	63	1	28	64	4
Beta-carotene	23	64	2	23	57	8
Lycopene	29	65	1	22	61	3

Vitamin C	21	53	9	20	53	6

^1^FFQ, food frequency questionnaire.

^2^For vitamin C, women (n=99).

Table 10 and Table 11 show the cumulative % contribution of the top 20 foods for alpha-carotene and beta-carotene assessed by DR. Carrots and tomato contributed more than 92% of the total alpha-carotene intake in both sexes. Carrots were the greatest contributor, and many kinds of leafy green vegetables were important contributors of beta-carotene.

**Table 10.** Cumulative % contribution of the top 20 foods for alpha-carotene assessed by DR

Food code^1^	Description^1^	*µ*g/day	Cumulative^2^ percent
Men (n=102)			
12-94a	Carrot/Root/Raw	400.1	88.1
12-85	Tomatoes/Fruit	19.1	92.3
12-19a	Leaf mustard/stems and leaves/Raw	13.8	95.4
13-79	Mangos/Raw fruit	5.5	96.6
12-92a	Bitter gourd/Fruit/Raw	3.9	97.4
12-6a	Kidney beans,Snap beans/Pods,immature/Raw	2.3	97.9
13-64	Bananas/Raw fruit	1.8	98.3
12-19b	Leaf mustard/stems and leaves/Boiled	1.4	98.6
13-17b	Satsuma mandarins/Raw fruit,sections with membranes/Normal ripening type	1.2	98.9
13-26a	Kaki,Japanese Persimmons/Raw fruit/Hard type	1.0	99.1
13-88	Apples/Raw fruit	0.9	99.3
12-83a	Maize,Corn/Sweet com,immature/Raw	0.7	99.5
12-35a	Sweet pepper/Fruit/Raw	0.6	99.6
12-84b	Maize,Corn/Cainned/Whole-kernel style	0.4	99.7
16-20	Green tea/Maccha,finely ground	0.2	99.7
12-36	Perilla/Leaves	0.2	99.8
13-24b	Oranges/Raw fruit,sections without membranes/Valencia	0.2	99.8
13-80b	Melons/Raw fruit/Hybrid melons	0.2	99.9
13-54	Natsumikan,Japanese summer orange/Raw fruit,sections without membranes	0.2	99.9
13-50	Tangors/Raw fruit	0.1	99.9
Women (n=113)			
12-94a	Carrot/Root/Raw	369.8	86.3
12-85	Tomatoes/Fruit	25.9	92.4
12-19a	Leaf mustard/stems and leaves/Raw	8.7	94.4
13-79	Mangos/Raw fruit	7.1	96.1
12-92a	Bitter gourd/Fruit/Raw	2.8	96.7
12-6a	Kidney beans,Snap beans/Pods,immature/Raw	2.1	97.2
13-64	Bananas/Raw fruit	1.9	97.6
13-17b	Satsuma mandarins/Raw fruit,sections with membranes/Normal ripening type	1.7	98.0
12-19b	Leaf mustard/stems and leaves/Boiled	1.7	98.4
13-26a	Kaki,Japanese Persimmons/Raw fruit/Hard type	1.3	98.7
13-88	Apples/Raw fruit	1.1	99.0
12-83a	Maize,Corn/Sweet corn,immature/Raw	1.0	99.2
16-20	Green tea/Maccha,finely ground	0.6	99.3
12-84b	Maize,Corn/Canned/Whole-kernel style	0.5	99.4
13-24b	Oranges/Raw fruit,sections without membranes/Valencia	0.4	99.5
12-35a	Sweet pepper/Fruit/Raw	0.4	99.6
13-54	Natsumikan,Japanese summer orange/Raw fruit,sections without membranes	0.3	99.7
12-36	Perilla/Leaves	0.2	99.8
13-80b	Melons/Raw fruit/Hybrid melons	0.2	99.8
13-27	Kaki,Japanese Dried fruit	0.2	99.8

^1^Food codes and descriptions correspond to those of the Standard Tables of Food Composition, 4th revised edition in Japan by Science and Technology Agency.

^2^Data on subjects in Ishikawa PHC (14-day data) were counted twice for 28-day data.

**Table 11.** Cumulative % contribution of the top 20 foods for beta-carotene assessed by DR

Food code^1^	Description^1^	*µ*g/day	Cumulative^2^ percent
Men (n=102)			
12-94a	Carrot/Root/Raw	1138.6	39.2
12-117a	Spinach/Leaves/Raw	355.1	51.5
12-93a	Chinese chive/Leaves/Raw	112.0	55.3
12-98b	Nozawana/Leaves/Salted	103.4	58.9
12-32a	Komatsuna/Leaves/Raw	92.6	62.1
12-18a	Pumpkin and squash/Squash/Raw	74.3	64.6
12-39a	Garland chrysanthemum/Leaves/Raw	57.5	66.6
12-85	Tomatoes/Fruit	49.3	68.3
12-55a	Daikon,Japanese radish/Leaves/Raw	43.9	69.8
12-77a	Basella/Leaves/Raw	33.1	71.0
13-79	Mangos/Raw fruit	31.1	72.0
12-74a	Chingentsuai/Leaves/Raw	27.4	73.0
12-72	Lettuce/Head lettuce,butter head type	26.0	73.9
12-114a	Broccoli/Head/Raw	26.0	74.8
12-19a	Leaf mustard/stems and leaves/Raw	25.6	75.7
13-45	Watermelon/Raw fruit	23.5	76.5
12-76	New Zealand spinach/stems and leaves	23.2	77.3
12-25a	Cucumber/Fruit/Raw	22.6	78.1
12-113a	Chard,Swiss chard/Leaves/Raw	21.6	78.8
13-80b	Melons/Raw fruit/Hybrid melons	14.8	79.3
Women (n=113)			
12-94a	Carrot/Root/Raw	1052.4	41.4
12-117a	Spinach/Leaves/Raw	361.4	55.6
12-93a	Chinese chive/Leaves/Raw	102.7	59.6
12-18a	Pumpkin and squash/Squash/Raw	93.5	63.3
12-32a	Komatsuna/Leaves/Raw	92.5	66.9
12-98b	Nozawana/Leaves/Salted	86.9	70.3
12-85	Tomatoes/Fruit	66.7	72.9
12-39a	Garland chrysanthemum/Leaves/Raw	54.5	75.1
12-77a	Basella/Leaves/Raw	43.1	76.8
12-55a	Daikon,Japanese radish/Leaves/Raw	42.6	78.4
13-79	Mangos/Raw fruit	40.3	80.0
13-45	Watermelon/Raw fruit	29.0	81.2
12-114a	Broccoli/Head/Raw	28.7	82.3
12-74a	Chingentsuai/Leaves/Raw	26.6	83.3
12-113a	Chard,Swiss chard/Leaves/Raw	24.1	84.3
12-25a	Cucumber/Fruit/Raw	22.5	85.2
12-72	Lettuce/Head lettuce,butter head type	21.9	86.0
12-76	New Zealand spinach/stems and leaves	19.3	86.8
13-80b	Melons/Raw fruit/Hybrid melons	18.6	87.5
13-17b	Satsuma mandarins/Raw fruit,sections with membranes/Normal ripening type	18.6	88.2

^1^Food codes and descriptions correspond to those of the Standard Tables of Food Composition, 4th revised edition in Japan by Science and Technology Agency.

^2^Data on subjects in Ishikawa PHC (14-day data) were counted twice for 28-day data.

## DISCUSSION

In the present study, we examined the correlation between dietary alpha-carotene, beta-carotene, lycopene and vitamin C intakes estimated from FFQ and DR. We also examined the possible correlation between the carotenoid and vitamin C intake and blood levels of carotenoid and vitamin C. Both in men and women, dietary alpha-carotene, beta-carotene, and vitamin C intakes estimated from FFQ were significantly correlated with intake from DR. However, the association between dietary lycopene intakes estimated from FFQ and DR was weak. Dietary carotenoid intake was associated with serum carotenoid level in men. However, there was no association in women except for alpha-carotene. Both in men and women, no significant correlation was observed for the plasma ascorbic acid level.

The correlation between dietary alpha- or beta-carotene intake estimated from the FFQ and DR was moderate. The alpha-carotene correlation coefficient was within the range of reported correlations (0.34-0.49) in earlier studies as was the beta-carotene correlation coefficient (0.20-0.50).^13^^,^^14^ Because carotenoids are not energy-provided nutrition, the correlation between carotenoid intakes estimated from FFQ and both intake from DR and serum carotenoid level was not improved when expressed as an energy-adjusted value.

The correlations between carotenoid intake estimated from FFQ and the corresponding serum carotenoid levels were higher in men than in women. Also, in men, correlations between carotenoid intake estimated from FFQ and corresponding serum carotenoid levels were higher than between intakes from DR and the corresponding serum carotenoid levels. In many previous studies, the correlations between dietary alpha-carotene intakes and the corresponding serum or plasma levels ranged from 0.25 to 0.53, while dietary beta-carotene intakes and the corresponding serum or plasma levels ranged from 0.15 to 0.36.^13^^-^^19^ Our observed correlation for men was within the range of these reported correlations. These results suggest that the data on carotenoid intake assessed with FFQ used in the JPHC study might be valid for men. However, it seemed reasonable to think that the bioavailability of carotenoids was different in men and women.

A low correlation was observed between dietary lycopene intake estimated from FFQ and those estimated from DR. This might be due to the inadequate portion size of tomatoes which contribute to more than 50% for the cumulative percentage of lycopene intake. In addition, because the lycopene database was quotation from the Department of Agriculture (USDA) food composition tables,^20^ it may be difficult to estimate lycopene intake in Japanese.

No meaningful correlation was observed between Vitamin C intake estimated from both DR and FFQ and plasma vitamin C levels. It is considered that vitamin C intake may vary in response to the availability of seasonal foods, and also the plasma vitamin C levels do not reflect habitual intake because of its short half-life. In addition, the estimated vitamin C intake in this study did not take account of cooking loss, and this is another reason for no meaningful correlation.

In summary, we observed a moderate correlation between dietary carotenoids and vitamin C intake estimated from the FFQ and DR. A moderate correlation was also found between dietary carotenoid intake and the corresponding serum carotenoid level for men; the correlation, however, was weak for women except for alpha-carotene. No significant correlation was observed with the plasma ascorbic acid level. These results suggest that carotenoid intake estimated from the FFQ is more valid in men than in women.
